# Systemic Sclerosis Pathogenesis and Emerging Therapies, beyond the Fibroblast

**DOI:** 10.1155/2019/4569826

**Published:** 2019-01-23

**Authors:** Andrea Sierra-Sepúlveda, Alexia Esquinca-González, Sergio A. Benavides-Suárez, Diego E. Sordo-Lima, Adrián E. Caballero-Islas, Antonio R. Cabral-Castañeda, Tatiana S. Rodríguez-Reyna

**Affiliations:** ^1^Department of Immunology and Rheumatology, Instituto Nacional de Ciencias Médicas y Nutrición Salvador Zubirán, Mexico City, Mexico; ^2^Tecnologico de Monterrey, Escuela de Medicina y Ciencias de la Salud, Mexico City, Mexico; ^3^Department of Rheumatology, University of Ottawa, Canada

## Abstract

Systemic sclerosis (SSc) is a complex rheumatologic autoimmune disease in which inflammation, fibrosis, and vasculopathy share several pathogenic pathways that lead to skin and internal organ damage. Recent findings regarding the participation and interaction of the innate and acquired immune system have led to a better understanding of the pathogenesis of the disease and to the identification of new therapeutic targets, many of which have been tested in preclinical and clinical trials with varying results. In this manuscript, we review the state of the art of the pathogenesis of this disease and discuss the main therapeutic targets related to each pathogenic mechanism that have been discovered so far.

## 1. Introduction

Systemic sclerosis (SSc) is a systemic autoimmune disease in which inflammation and fibrosis play a crucial role and lead to severe damage and failure of multiple organs such as the skin, joints, tendons, gastrointestinal tract, lungs, heart, blood vessels, and kidneys. It primarily affects women (female:male ratio of 4:1-10:1, depending on age and ethnicity) [1, 2], and there are 2 clinical subsets according to the extent of skin involvement: diffuse cutaneous SSc (dcSSc) (skin damage proximal to elbows and/or knees or that affects thorax and/or abdomen at any given time during the disease) and limited cutaneous SSc (lcSSc) (skin damage distal to elbows and knees without involvement of either thorax or abdomen). This disease may lead to major disabilities due to vascular complications, cardiopulmonary involvement, inflammatory myopathy, and arthritis; likewise, it can cause malnutrition due to gastrointestinal tract involvement, and it can decrease quality of life as a consequence of the psychological and social impact. Additionally, it can be fatal, with a 3-year survival rate of 47-56% in cases of serious pulmonary or cardiac involvement, particularly PAH [3–5]; in fact, it is the single connective tissue disease with the worst survival prognosis [1].

Groups of experts have tried to come to a consensus regarding treatment for specific organ involvement. Such is the case of EULAR's recommendations for the treatment of systemic sclerosis, which aims to guide treatment for patients based on evidence and clinical experience from worldwide experts; however, there is still no standardized and effective treatment for this disease to date [2].

The understanding of the pathogenesis of the disease has improved considerably in recent years. Although there are still many unanswered questions, the participation of the immune response cells and inflammatory mediators, fibroblasts, and other components of the extracellular matrix and the central role of endothelial damage have changed the paradigm of this disease that was previously considered as predominantly fibrotic. Now it is conceived as a complex syndrome with multiple pathogenic pathways that may be treated simultaneously.

In recent years, there has been substantial progress in the management of some complications developed by these patients, which has led to increased disease survival and quality of life. This includes better control of complications in specific organs (such as interstitial lung disease, pulmonary arterial hypertension, renal crisis, and Raynaud's phenomenon) as well as standardized follow-up and earlier detection of potential complications [6].

The ideal of “targeted therapy” will be an increasingly attainable objective insofar as our understanding of the disease improves. As stated by Dr. Denton [4], this concept in systemic sclerosis can have different meanings: the first one refers to the treatment of specific organ complications such as renal crises, interstitial lung disease, and pulmonary arterial hypertension; the second one refers to the treatment of symptoms of a determined organ involvement such as Raynaud's phenomenon or gastroesophageal reflux; the third one refers to the treatment of individual disease processes such as immune activation, inflammation, fibrosis, or vasculopathy; and the last one refers to the blocking of certain cell types or interactions between cells that impact on various aspects of the disease through the same mechanism, even and ideally blocking an intracellular target or a specific pathway that modifies several pathological processes of the disease. Many molecules involved in the pathogenesis of this disease have been evaluated as therapeutic targets in preclinical and clinical trials with diverse outcomes (Tables 1 and 2) [7]. Here we present the state of the art of the pathogenic pathways and proposed targeted therapies.

To perform this literature review, we conducted a research through electronic resources (PubMed, ScienceDirect, Nature, Elsevier, BMJ, and Wiley Online), reviewing references in the English language from the last 10 years. We identified articles via general search of the terms “systemic sclerosis OR scleroderma” and “systemic sclerosis pathogenesis;” the first search yielded 6334 articles, which were handpicked according to relevance that was determined according to the article's date of publication, ranging from 2008 to October 2018, and its direct relation to scleroderma pathogenesis and directed therapies. Subsequently, we directed a specific search of the terms “Bosentan”, “Macitentan”, “Ambrisentan”, “Selexipag”, “Riociguat”, “bardoxolone methyl”, “Infliximab”, “Adalimumab”, “Rituximab”, “Basiliximab”, “Efalizumab”, “Abatacept”, “AIMSPRO”, “Tocilizumab”, “AM095”, “SAR100842”, “Imatinib”, “Dasatinib”, “Nilotinib”, “CAT-192”, “GC-1008”, “FG-3019”, “P144”, “*α*v*β*6 integrin”, “Pirfenidone”, and “Nintedanib”, which resulted in a range of 2 to 300 references per term.

## 2. Etiopathogenesis of Systemic Sclerosis and Therapeutic Targets

**Figure 1:**
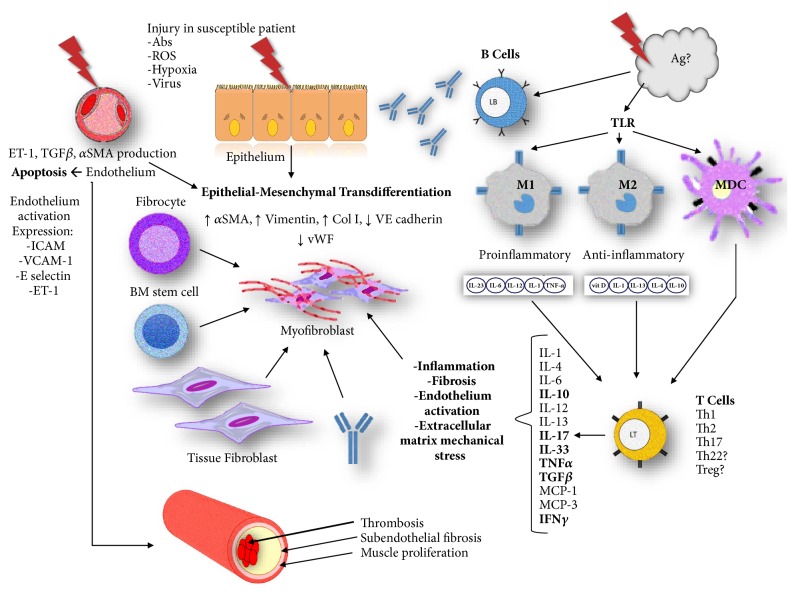
Scheme of the pathogenesis of systemic sclerosis. Participation of the immune system, epithelium, endothelium, and fibroblasts. Theoretically, an unknown self or foreign antigen (Ag) starts an autoimmune response in a susceptible individual, producing damage to endothelial and/or epithelial cells. The abnormal activation of the innate and adaptive immune system leads to the production of proinflammatory and profibrotic cytokines and to autoantibody production. Endothelial cells may undergo apoptosis, activation, or endothelial to mesenchymal transdifferentiation, while epithelial cells may undergo either epithelial to mesenchymal transdifferentiation or inflammation and injury. Then, myofibroblasts recruited from different sources (fibrocytes, bone marrow stem cells, tissue fibroblasts, or endothelial/epithelial transdifferentiation) concentrate at the extracellular matrix and produce excessive fibrosis that leads to organ damage. In addition, blood vessel injury promotes in situ thrombosis, subendothelial fibrosis, and muscular proliferation, leading to the vascular manifestations of the disease.** ET-1:** endothelin 1,** TGFβ:** transforming growth factor beta,** TNFα:** transforming growth factor alpha, **αSMA:** alpha smooth muscle actin,** Ab:** antibodies,** ROS:** reactive oxygen species,** ICAM:** intercellular adhesion molecules,** VCAM-1:** type 1 vascular cell adhesion molecules,** Ag:** antigens,** Col 1:** collagen type 1,** VE:** vascular endothelial,** vWF:** Von Willebrand factor,** BM:** bone marrow,** TLR:** Toll-like receptor,** M1:** type 1 macrophage,** M2:** type 2 macrophage,** MDC:** myeloid dendritic cell,** IL:** interleukin,** IFNγ:** interferon gamma,** MCP-1:** monocyte chemoattractant protein type 1,** MCP-2: **monocyte chemoattractant protein type 2,** Th:** T helper lymphocyte, and** Treg:** T regulator lymphocyte.

The triggering factors that unleash the pathogenic processes that lead to the development of systemic sclerosis have not been yet identified. The most accepted working hypothesis implies that, in a genetically susceptible subject, a triggering event occurs (probably exposure to an infectious or environmental agent such as vinyl chloride or silica, or an event that supposes an important immunological challenge such as a pregnancy or the development of a neoplasia), which causes the activation of various cells of the immune system, the endothelium, and the extracellular matrix. These cells then lead to inflammation, fibrosis, and endothelial damage that cannot be counterbalanced by the natural resolution mechanisms of inflammatory processes and scarring (Figure 1).

### 2.1. Involvement of the Immune System

The most acknowledged evidence of the participation of the immune system in this disease is the presence of various autoantibodies, several of which are present exclusively in this disease and are associated with clinical complications and specific phenotypes as has been broadly described by Dr. Thomas A. Medsger Jr. and his work team [1].

Furthermore, several genetic studies have found, as additional evidence, an association of different gene polymorphisms related to the immune response with the predisposition to suffer systemic sclerosis. Some of the most important ones are polymorphisms in genes of the major histocompatibility complex [8], in regulatory genes of types I and II interferons, in genes of cytokines and chemokines, and of Toll-like receptors (TLR) [9].

Skin from SSc patients shows inflammatory infiltrates in which macrophages, T lymphocytes, and dendritic cells are the predominant cell types [31]. These cells produce cytokines and chemokines with proinflammatory and profibrotic activities, and it is very possible that they participate in the process of endothelium-mesenchymal and epithelial-mesenchymal transition, processes by which endothelial and epithelial cells are activated and acquire characteristics similar to myofibroblasts [10]. The powerful signals generated by cytokines and chemokines also produce the recruitment of bone marrow cells and fibrocytes from the peripheral circulation for their subsequent activation into fibroblasts that will produce collagen and other extracellular matrix proteins that predominate in the fibrotic phase of this disease (Figure 1).

#### 2.1.1. Innate Immune System

Although the factors that promote the persistent activation of cells of the immune system are unknown, recent studies of various groups have pointed towards the Toll-like receptors as possibly responsible for interacting with their classical agonists or with other exogenous or endogenous agonists from damaged tissue to activate dendritic cells, which could secrete proinflammatory cytokines and present antigens to the T cells to activate them. Overexpression of TLR4 and TLR2 has been found in skin and fibroblasts of patients with SSc [32, 33]. On the other hand, the TLR3, TLR7, TLR8, and TLR9, generally viral nucleic acid sensors, could be involved in inflammation in systemic sclerosis; Farina et al. [10] demonstrated the association between Epstein-Barr virus infection, overexpression of interferon-associated genes, transforming growth factor-beta (TGF*β*), and other markers of fibroblast activation. In this sense, persistent damage after a viral infection could cause chronic inflammation and fibrosis in susceptible subjects. Activation of dendritic cells through TLRs generally leads to the production of several proinflammatory cytokines, particularly type I interferons, which have been found overexpressed in peripheral blood in patients with systemic sclerosis (interferon signature) [34].

In fact, nearly 50% of SSc patients show the so-called “interferon signature” in peripheral blood and sera [35]. These abnormalities are seen in some SSc patients even at early phases of the disease, before skin fibrosis is well established. In this group of patients, the type I IFN signature in peripheral monocytes correlates with inflammation and fibrosis mediators (B cell activating factor (BAFF) expression and type III procollagen N-terminal propeptide serum levels) [36], which suggests an association between abnormal activation of the IFN-I signaling pathway and disease activity [37].

The consequences of increased type I IFN expression in SSc are diverse and affect immune and endothelial cell function as well as fibroblasts. These effects have been extensively reviewed by Ciechomska and Skalska [38] and are outlined below.

IFN type I effects on immune cells include increased monocyte activation, as well as increased differentiation, survival, proliferation, and activation of T, B, and dendritic cells [10, 36, 38–41]. Moreover, Kim et al. showed that serum of SSc patients containing anti-topoisomerase I antibodies induces the production of interferon-alpha by PBMCs cocultured with nuclear extracts. This was particularly higher when they used serum from dcSSc patients and from SSc patients with lung fibrosis [42].

On the other hand, type I IFN effects on endothelial cells include the increased expression of MxA (marker of type I IFN activity) and interferon regulatory factors (IRGs), which correlate with the presence of digital ischemic ulcers [43, 44]. Also, human dermal microvascular endothelial cells (HDMVECs) and fibroblasts stimulated with IFN*α* and IFN*γ* show increased vessel permeability, increased expression of alpha smooth muscle actin (*α*-SMA), connective tissue growth factor (CTGF), transforming growth factor beta 2 (TGF-*β*2), and endothelin 1 (ET-1), via downregulation of friend leukemia integration 1 transcription factor (Fli1) and downregulation of vascular endothelial cadherin (VE-cadherin) [45].

Finally, type I IFN stimulates the expression of TLRs on DCs and fibroblasts; this leads to increased inflammatory cytokine production by fibroblasts. For instance, type I IFN induces fibroblasts to increase IP-10 production, a profibrotic chemokine that has been associated with severe SSc subtypes [46, 47]. Additionally, positive feedback has been demonstrated between type I IFN and TLR expression, since TLR3 stimulation with its ligand poly I:C also induces upregulation of type I IFNs and IFN*α*2 responsive genes on fibroblasts [48, 49].

It is also known that the number of plasmacytoid dendritic cells (pDCs) is increased in the circulation of SSc patients, and they secrete large amounts of CXCL4 [50], a chemokine associated with transition from epithelial to mesenchymal cells, the activation of endothelial cells, inhibition of regulatory T cells, and induction of Th17 cells.

On the same line, a murine model showed that IL-33, an alarmin of the IL-1 family related to inflammation and fibrosis, favours IL-13-dependent lung fibrosis [51]. Another group showed that TLR4-deficient mice developed less tissue fibrosis, decreased polarization of Th17 responses, and decreased TGF-*β* in the bleomycin-induced fibrosis and the tight skin models [52]. It is unknown whether IL-33 can induce fibrosis by this or another pathway in SSc patients.

One of these pathways could be the production of profibrotic cytokines by type 2 innate lymphoid cells. Proliferation and function of these cells are stimulated by exposure to cytokines with epithelial alarmin function such as IL-33 and IL-25, both elevated in patients with systemic sclerosis, and they can produce profibrotic cytokines (IL-4 and IL-13), so they could participate in the pathogenesis of this disease. There is controversial evidence on the abundance and relevance of these cells in patients with systemic sclerosis [53, 54]; however, in a murine model of pulmonary fibrosis it was found that the IL-13 derived from these cells increases the deposit of collagen by the fibroblasts and induces the differentiation of macrophages towards a profibrotic phenotype [55].

Evidence of the involvement of macrophages in the pathogenesis of systemic sclerosis is extensive. Infiltrates of CD68+ cells (macrophage marker) CD163+, CD204+ and an M2 macrophage markers panel were found in the perivascular regions and between the collagen fibers of patients with SSc. Increased CD14+CD163+CD204+ cells have also been found in peripheral blood of patients with SSc, as well as increased markers of macrophage migration and activation (CCL18 and CD163) in microarrays of lung tissue from patients with progressive pulmonary fibrosis [41]. Macrophages can also be stimulated through TLRs and their activation, particularly that of so-called M2 or “alternatively activated” macrophages, would lead to the production of profibrotic substances such as IL-13, TGF*β*, platelet-derived growth factor (PDGF), and chemokines such as CCL19 which stimulates the activation of macrophages.

#### 2.1.2. Adaptive Immune System

T lymphocytes are also found in inflammatory infiltrates in tissues of patients with SSc; they display higher expression of activation markers and there is evidence indicating that they express a rather oligoclonal repertoire of T cell receptors, suggesting an antigen-mediated expansion [56]. Th2 cells, producing IL-4 and IL-13, and Th17, producing IL-17, have been found to be increased in both skin and peripheral blood of patients with SSc, particularly in patients with the diffuse form of the disease. The cytokines they produce have important profibrotic and proinflammatory functions; it is likely that these cells are activated by antigen-presenting cells such as dendritic cells [57]. The role of regulatory T cells is less clear since several studies have shown controversial results, but given that it is a very small population of cells, it is possible that its function and regulation, rather than its number, is what is found altered in this and other autoimmune diseases.

B cells, on the other hand, are the producers of the autoantibodies characteristic of this disease, but we also know that these cells infiltrate tissues and show increased activation markers such as CD19, CD21, costimulatory molecules, and B cell activating factor (BAFF). There is evidence in murine models that overexpression of CD19 induces the production of cutaneous fibrosis and that the absence of B cells is associated with decreased fibrosis [58].

### 2.2. Targeted Therapies for Inflammatory Process

#### 2.2.1. Type I Interferon Modulation

Interferons are pleiotropic cytokines that play a fundamental role as factors responsible for the immune response, mainly in bacterial and viral infections; however, they have also been strongly associated with the pathogenesis of SSc because of their notorious correlation with skin thickness and disease activity when found in elevated levels in patients' blood and sera [38]. Consequently, several clinical trials have been executed in order to test the potential benefits on directed anti-IFN treatments, as follows:

(*1) Anifrolumab   ***→***  Type I Interferon Receptor. *Displaying promising results, anifrolumab is an investigational human IgG1*κ* monoclonal antibody that has been tested on a phase I trial to treat SSc [59]. According to Peng* et al.*, this drug blocks the formation of the ternary IFN/IFNAR1/IFNAR2 signaling complex by sterically inhibiting the binding of IFN ligands to IFNAR1 [60]. At the same time, regarding SSc patients, this antibody's safety profile is considered favorable because of the mild to moderate adverse events triggered by it, which include upper respiratory tract infection, headache, diarrhea, and nausea [61].

*(2) MEDI7734* **→** *Anti-ILT7. *Recently AstraZeneca conducted a phase I study of MEDI7734 on a human monoclonal antibody that binds to and causes temporary depletion of plasmacytoid dendritic cells (pDCs) in which the safety, drug levels, and pDC levels in patients with type I IFN-mediated autoimmune diseases were evaluated. pDCs are one of the main type I IFN sources [62]. No results have been published yet.

#### 2.2.2. Rituximab → CD20

Rituximab is a chimeric monoclonal antibody (mAb) that targets CD20, which is expressed from pre-B cell stage to the pre-plasma cell stage [63]. In systemic sclerosis, there is evidence that suggests this drug has an antifibrotic effect [64], as well as potential to improve inflammatory alterations [65] and lung function [66], which characterize several SSc manifestations.

According to Giuggioli's literature review, after six months of rituximab treatment in patients with either lcSSc or dcSSc, there was clear improvement of both articular and skin SSc manifestations, as well as a safety profile and tolerance; more specifically, it has been documented that the number of swollen and tender joints was markedly reduced after treatment, skin sclerosis improved significantly (specially in patients with diffuse cutaneous involvement), and, similarly, other skin and joint manifestations mitigated, such as melanodermia, pruritus, calcinosis, and arthritis [65].

#### 2.2.3. Basiliximab → IL-2R*α*

Basiliximab is a chimeric mAb directed against the *α* chain (CD25) of the IL-2 receptor that has recently been proposed for the treatment of SSc based on the latest discoveries regarding the crucial role of effector T cells in this disease, particularly Th-17 and T regulatory subsets [67].

As stated by Schmidt* et al.*, skin involvement, lung fibrosis disease progression, and mortality in systemic sclerosis could be ameliorated by the treatment with this drug, since they are strongly correlated with serum levels of soluble IL-2 receptor [68]; regarding side-effects, the ones recorded are mostly minor and, in general, therapy with basiliximab was well tolerated in an open-label SSc study [11].

#### 2.2.4. Efalizumab → LFA1/ICAM-1

The binding of leucocyte function associated antigen 1 (LFA-1) to intracellular adhesion molecule 1 (ICAM-1) is a key step in the migration of T lymphocytes through the endothelial lining of the vascular system during inflammation in skin disorders [69]. In the presence of efalizumab, a humanized recombinant IgG1 monoclonal antibody, the *α*-subunit of LFA-1 (CD11a) is targeted; thus, the interaction between LFA-1 and ICAM-1 is blocked, hence hindering T-cell's activation, migration into the skin, and cytotoxic functions [12]. This mechanism of action seems attractive for systemic sclerosis treatment, since increased numbers of T lymphocytes are usually found in dermal infiltrates in this disease. Currently, efalizumab is approved to ameliorate the size and severity of skin lesions in patients with psoriasis, and it has shown sustained long-term response [70]. No SSc clinical trials have been published.

#### 2.2.5. Abatacept → CTLA4

Abatacept is a recombinant CTLA4Ig fusion protein that inhibits T-cell activation by selectively modulating costimulation [13] as it binds to CD80 or CD86 on the T-cell surface.

It has been approved for the treatment of arthritis [71], and it is proposed that its effects on inhibition of T-cell activation may be efficacious in dcSSc [67]. There is also evidence that suggests that this drug could be safe and effective in patients with refractory polyarthritis secondary to scleroderma [13, 72].

#### 2.2.6. AIMSPRO (®) → *α*MSH, IL10, CCL2

Otherwise known as hyperimmune caprine serum, AIMSPRO is a polyclonal antibody that contains mainly caprine immunoglobulins as well as cytokines, including IL-4 and IL-10, proopiomelanocortin, arginine vasopressin, *β*-endorphin, and corticotropin-releasing factor [14]. This drug could potentially modulate serum levels of relevant cytokines.

Results in Quillinan's trial on AIMSPRO treatment in scleroderma showed potential benefit in skin tightness in late cases as well as improvement in overall pain, which is presumably of clinical importance since pain related to tissue ischaemia, inflammation, and intermittent release of neuropathic mediators, presumptively. The drug's safety profile was adequate, and it was well tolerated [14].

#### 2.2.7. Tocilizumab → IL-6R

Tocilizumab is a monoclonal antibody to the IL-6 receptor [71] that has been tested for diverse SSc clinical manifestations, since an increased production of IL-6 in fibroblasts isolated from the affected skin of SSc patients has been documented [73].

This pleiotropic cytokine has several significant roles in hematopoiesis, inflammation, and immune homeostasis, as well as in T-cell growth and differentiation; there is also evidence that elevated levels of IL-6 are present in other fibrotic diseases such as keloid scars and lung fibrosis, among others.

According to Ong et al., recent findings suggest that therapeutic intervention in fibrotic pathways could be viable by IL-6 modulation, in addition to being a useful tool to promote immune tolerance in systemic sclerosis because of its regulatory effect in the balance between Th17 and Tregs [67].

Regarding clinical effectiveness, there is evidence that this drug highly improves joint parameters after five months of treatment, as well as skin involvement [74]; it has been reported to be effective in refractory polyarthritis and myopathy [75]. Recent evidence in a phase 3 double-blinded clinical trial suggested mild skin improvement and stabilization of lung involvement [76].

#### 2.2.8. AM095 and SAR100842 → LPA_1_

Lysophosphatidic acid (LPA) is a phospholipid growth factor that targets specific G-protein-coupled receptors that have recently been associated with the pathogenesis of systemic sclerosis. It is generated at inflammation sites or cell damage via autotaxin on lysophosphatidylcholine and other lysophospholipids [77] and could possibly contribute to excessive tissue fibrosis, primarily through the activation of the LPA 1 receptor [78].

SAR100842 is a low molecular weight, orally available selective inhibitor of LPA 1 receptor that aims to ameliorate or even revert fibrotic progression in SSc [79]. According to Allanore's research, there is important mRSS score improvement after 24 weeks of treatment, which is of clinical significance; and it is an overall well tolerated drug in dcSSc patients, with mild to moderate in intensity adverse effects [79]. There is also evidence that SAR 100842 lowers expression of fibrosis-related genes in scleroderma skin fibroblasts [71].

#### 2.2.9. TAK242 → TLR4

As previously stated, TLR4 stimulation promotes the production of Th1 and Th17 cytokines, and increased levels of this molecule and its ligands have been found in SSc patients. Dr. Varga research group has elegantly shown that TLR4 inhibition with TAK242 prevents and induces regression of experimental fibrosis in bleomycin-induced fibrosis and in TSK/+ mice. His findings suggest that TAK242 might represent a therapeutic strategy for the treatment of SSc and other fibrotic diseases [15].

#### 2.2.10. Inebilizumab → Anti-CD19

Also referred to as MEDI-551, it is a humanized, affinity-optimized, and afucosylated monoclonal antibody that binds to CD19 [80]. In 2014 a phase I clinical trial regarding the safety and tolerability of this drug was completed, in which Schiopu et al. determined that B-cell depletion should be further studied because of its significance regarding the pathogenesis of the disease along with inebilizumab's pharmacodynamics, which could potentially become a highly beneficial disease-modifying treatment in SSc [81].

### 2.3. Fibrosis

Fibrosis is the most prominent clinical feature of systemic sclerosis and, largely, the process that leads to the deterioration of the organs' function affected by the disease. It occurs because of excess production of collagen and other extracellular matrix proteins in the connective tissue of various organs.

Myofibroblasts, the main cells responsible for the production of the extracellular matrix in this disease, can have different origins. It has been suggested that they may come from endothelial cells (endothelium-mesenchymal transdifferentiation), from epithelial cells (epithelial-mesenchymal transdifferentiation), from bone marrow stem cells, from circulating fibrocytes, from fibroblasts already resident in tissues, and from resident stem cells in skin and subcutaneous cellular tissue [82].

Endothelium to mesenchymal transdifferentiation has been elegantly studied by Dr. Sergio Jiménez and his group [82]. In summary, it is proposed that the endothelial cell of a susceptible subject would be subjected to some initial insult that could be the presence of autoantibodies, reactive oxygen species, hypoxia, viral antigens, or own neoantigens; this initial insult would cause the abnormal activation of endothelial cells, which would undergo a transformation that would lead them to express more alpha smooth muscle actin (*α*SMA), vimentin and type I collagen, and lower amount of cadherin and von Willebrand factor (vWF), converting them into collagen-producing cells similar to myofibroblasts (Figure 1).

The epithelium has a very important role in the repair of injuries; in patients with systemic sclerosis there is evidence that the process of epithelial regeneration is altered. We know that there are many factors derived from the epithelium that influence the behavior of fibroblasts; particularly endothelin 1 (ET-1) and TGF-*β* have profibrotic activity. There is evidence that the epithelial to mesenchymal transdifferentiation process occurs in pulmonary fibrosis and that both ET-1 and TGF-*β* participate in this process [83]. During epithelial-mesenchymal transdifferentiation, epithelial cells lose their intercellular junctions and change their polarity, different surface markers are expressed, and there may be remodeling of their cytoskeleton to express a mesenchymal phenotype. Some in vitro studies have shown that alveolar epithelial cells can be transdifferentiated to mesenchymal cells [84]. In addition, some studies in murine models have shown that alveolar epithelial cells can coexpress markers of both epithelial cells and mesenchymal cells, including cadherin and *α*-SMA [85]. Another evidence in this sense is that, in the murine model of pulmonary fibrosis induced by bleomycin, pulmonary fibrosis is preceded by epithelial damage. This evidence suggests that epithelial damage is important in the pathogenesis of systemic sclerosis, at least for some organs, such as in pulmonary fibrosis and, most likely, the skin.

Regardless of their origin, we know that fibroblasts have different functional phenotypes according to their location (dermis, subcutaneous cellular tissue, lungs, etc.) and can be distinguished by their gene expression profile and their functional activity. Depending on their microenvironment, they can produce different amounts of procollagen, fibronectin, proteases, collagenases, and other regulators of the extracellular matrix. For example, inactive fibroblasts express ET-1 and intracellular adhesion molecules 1 (ICAM-1), whereas fibroblasts exposed to mechanical stress in the microenvironment, a situation that occurs in systemic sclerosis, express *α*-SMA, TGF-*β*, and genes associated with the production of extracellular matrix proteins. This phenotype is like that of fibroblasts exposed to an excess of TGF-*β* signaling [86].

Likewise, it is known that during different phases of tissue repair after a lesion fibroblasts of different origin produce different amounts and types of collagen that influence this process and that could be altered in patients with SSc [87].

### 2.4. Targeted Therapies for Fibrosis

#### 2.4.1. Imatinib, Dasatinib, Nilotinib → c-Abl, c-Kit, PDGF

Imatinib is a tyrosine kinase inhibitor (TKI) capable of blocking both PDGF and TGF-*β* signalling pathways. It showed antifibrotic effects in SSc experimental models and then it was evaluated in small clinical trials [16]. Dasatinib and nilotinib, which are second-generation TKIs with higher affinity to Bcr-Abl, and their ability to block c-abl and PDGF were also evaluated for the treatment of dermal fibrosis* in vitro* and in murine models with promising results [17].

Initial studies showed that low-dose imatinib had an adequate safety profile and a better tolerability than at high doses in the long term for SSc patients [74]. Furthermore, while it had no significant effects on skin involvement in a phase II pilot study, it was effective when used to stabilize lung function in patients with SSc-ILD [88].

#### 2.4.2. CAT-192 → TGF*β*1

Also known as metelimumab, CAT-192 is a human recombinant IgG monoclonal antibody that specifically counteracts the TGF-ß1 isoform in SSc. Despite its potential benefit via the inhibition of TGF-ß1, a multicenter, randomized, placebo-controlled phase I/II trial using this drug proved no efficacy and significant side effects, including mortality in patients that received the active treatment [18].

#### 2.4.3. Fresolimumab → TGF*β*1,-*β*2,-*β*3

Otherwise called GC-1008, fresolimumab is a monoclonal antibody that, unlike metelimumab, can target all isoforms of TGF*β* and has yielded very promising results in SSc.

Patients treated with this drug generally display expeditious declines in mRSS scores and infiltration of myofibroblasts into the dermis, as well as in TGF*β*-regulated genes' expression [74]. Bleeding is the main side effect that was recorded in the initial trial [89].

#### 2.4.4. FG-3019 → CCN2

FG3019 is a specific IgG1*κ* monoclonal antibody to CTGF that has shown potential in decreasing lung fibrosis and scarring according to recent research; however, no specific trials have been conducted in SSc [19].

Treatment with FG-3019 is highly efficient in reducing the number of CD45-positive cells; it also has an antifibrotic effect similar to the genetic deletion of CTGF in collagen-producing cells, which ameliorates angiotensin II-induced skin fibrosis as well as inflammation [7].

#### 2.4.5. P144 → TGF*β*1

Peptide 144 is the acetic salt of a 14-mer peptide from human TGF*β*1 type III receptor that was precisely designed to block the interaction between TGF*β*1 and TGF*β*1 type III receptor, consequently inhibiting its biological effects [90].

This drug has shown important antifibrotic activity in mice receiving repeated subcutaneous injections of bleomycin; however, P144 is still undergoing investigations regarding the treatment of skin fibrosis in patients with SSc [20].

#### 2.4.6. Anti-Integrin *α*V*β*6 (Abituzumab) → TGF*β* Activation

The integrin *α*V*β*6 is a LAP-binding integrin, mostly expressed in epithelial cells adjacent to wounds [91], that is involved in the initiation of fibrosis and inflammation by TGF*β* activity. It promotes activation and differentiation of fibroblasts into myofibroblasts, which causes abnormal extracellular matrix deposition, leading to the destruction of tissue architecture, scarring, and reduced function [92]. Truncation of this integrin's cytoplasmic tail (associating with the cytoskeleton) prevents latent TGF-*β*1 activation, thus suppressing the fibrotic process [93].

More precisely, evidence from preclinical models of lung, kidney, and liver fibrosis proposes that inhibition of *α*V*β*6-mediated TGF-*β* activation could potentially be useful to attend to multiple fibrotic disorders in humans [91] since specifically targeting *α*v*β*6 may reduce the risk of interfering with the beneficial homeostatic control of inflammation and immunity in the treatment of tissue fibrosis [94].

No clinical studies have been completed in this pathway at this time. However, it has been reported by Henderson et al. that V integrins collectively regulate the key profibrotic pathways during organ fibrosis [21]. Indeed, overexpression of integrin *α*V consequently leads to TGF-*β* overactivation in SSc dermal fibroblasts because of miR-29's involvement as a modulator of integrin's genes implicated in this pathway, as well as miR-142-3p, which directly regulates the expression of integrin *α*V, as stated by Li et al. [95], whose work is supported by Taniguchi's epigenetic study on bleomycin-induced skin fibrosis Fli-1 +/- mice, which proved latent TGF-*β* activation to be an *α*V*β*3 integrin- and *α*V*β*5 integrin-dependent mechanism [96].

Regarding directed therapies on this pathway, abituzumab is a novel, humanized monoclonal IgG2 antibody to the *α*v subunit that inhibits binding to *α*v heterodimers, preventing ECM attachment, cell motility, and apoptosis, without cross-reacting with other integrins, which is elemental in inhibiting TGF-*β*, a key mediator of fibrosis [97]. According to the Clinical Trials registry, there was an ongoing randomized clinical trial on this drug which was recently terminated due to difficulties in enrolling subjects under the eligibility criteria, not allowing for completion of the study within a reasonable time-frame [98].

#### 2.4.7. Pirfenidone → TNF*α*, IL1*β*, TGF*β*

Pirfenidone is an orally active pyridone small molecule with known anti-inflammatory, antifibrotic, and antioxidant properties that has proven to reduce fibroblast proliferation and block TGF-*β*-stimulated collagen synthesis; it has been approved for the treatment of mild to moderate idiopathic pulmonary fibrosis, disease in which it was associated with modest improvement in function (IPF) [22].

According to Xiao, pirfenidone attenuates lung fibrosis by interfering with the hedgehog signaling pathway in SSc-ILD lung fibroblasts [99]; and consistent with the LOTUSS trial, it has an acceptable tolerability and safety profile in patients with SSc-IP [23]. Scleroderma Lung Study III, a pirfenidone clinical trial for SSc-ILD, is currently ongoing, and it will shed light on the possible indication of pirfenidone for this disease.

#### 2.4.8. Nintedanib → VEGF, PDGF, FGF

Nintedanib, also known as BIBF 1120, is a TKI targeting fibroblast growth factor (FGF) receptor, PDGF receptor, and vascular endothelial growth factor (VEGF) receptor, as well as Src-family tyrosine kinases [71], that is characterized by its broad spectrum of profibrotic targets, which likely offers additive effects as compared with selective inhibition of individual profibrotic molecules [100].

As stated by Varga et al., there is evidence that this drug reduces dermal microvascular endothelial cell apoptosis and modulates the pulmonary vascular restoration by its effect on the number of vascular smooth muscle cells [73]; similarly, it has been confirmed to block proliferation and transformation of human lung fibroblasts, as well as collagen synthesis in skin fibroblasts from SSc patients [58]. A clinical trial involving this molecule is currently ongoing (SENSCIS study) [24].

### 2.5. Vasculopathy

Raynaud's phenomenon and vasculopathy associated with it, renal crises, and pulmonary arterial hypertension (PAH) are the classic vascular manifestations of scleroderma. Without a doubt, the endothelium plays a very important role in the initiation and perpetuation of vascular damage in this disease.

It is known that the endothelial damage that occurs from early stages in SSc could lead to 3 paths:

1. Endothelial cell apoptosis, which can lead to blood vessel destruction, that decreased blood flow seen as capillary loss in capillaroscopy
2. Endothelial to mesenchymal transdifferentiation explained previously
3. Endothelial cell “activation,” which refers to overexpression of chemotactic and vasoconstrictor substances such as ICAM-1, vascular adhesion molecule 1 (VCAM-1), E-selectin, and endothelin-1 (ET-1) on endothelial cell surface, leading to vasoconstriction and subendothelial fibrosis, as this process contributes to the development of intraluminal thrombosis and proliferation of the muscular layers, typical characteristics of vasculopathy in systemic sclerosis [82]

Another prominent process in systemic sclerosis, as a reaction to the loss of blood vessel function and hypoxia, is neoangiogenesis. In SSc patients, angiogenesis is abnormal. Angiogenic factors such as PDGF, VEGF and its receptors, ET-1, TGF-*β*, the monocyte chemoattractant protein 1 (MCP-1), and the urokinase-type plasminogen activator receptors are upregulated, despite the lack of adequate angiogenic responses in ischemic tissues in patients with SSc [101]. Likewise, counter-regulatory factors such as angiostatin and endostatin are persistently increased. The regulation of these systems in patients with SSc is not completely understood.

The generalized vasculopathy in this disease has 2 variants of particular interest: the thrombotic microangiopathy that develops during renal crisis and is histologically indistinguishable from the changes produced in malignant hypertension and the plexiform lesions produced in the advanced phases of pulmonary arterial hypertension.

### 2.6. Targeted Therapies for Vascular Damage

#### 2.6.1. Bosentan, Macitentan → ET_A_/ET_B_ Receptor

PAH has been treated for a long time now by using either bosentan or macitentan, two currently approved ERAs, which block both ET_A_ and ET_B_ endothelin receptors that mediate the detrimental effects of ET-1 in this particular disease in which the ET pathway plays a very important role [102].

Bosentan has higher affinity towards ET_B_ receptors and essentially the same affinity for the ET_A_ receptors, and it occupies the orthosteric site of the receptor to block the action of ET-1 by sterically preventing the inward movement of transmembrane helix six of the ET_B_ receptor [103], mechanism that is expected to be preserved in the ET_A_ subtype. Aside from PAH, it has also been proven to reduce the number of new digital ulcers, even in patients with multiple ones, regardless of usage of calcium channel blockers, PDE-5 inhibitors or iloprost therapy, having a highly evident effect in patients with four or more digital ulcers at baseline in the RAPIDS-2 trial. Its effectiveness indicated no difference in either of the disease's subsets [25].

On the other hand, macitentan was designed to have improved efficacy and higher potency and selectivity over bosentan, and according to Davenport* et al.*, that advantage is due to a longer receptor occupancy [26, 103]. Additionally, pharmacokinetic data have demonstrated that macitentan and its active metabolite both have a long elimination half-life of approximately 16 and 48 hours, respectively, which supports a once-daily dosing regimen [104]. It has been approved in more than 55 countries for the long-term treatment of patients with PAH as monotherapy or in combination with other PAH therapies, as was studied in the SERAPHIN Trial [26].

There have also been several studies regarding both drugs' efficacy on the fibrotic component of the disease, more specifically, pulmonary fibrosis with modest results [105].

#### 2.6.2. Ambrisentan → ET_A_ Receptor

Ambrisentan, which is meant to block the action of endothelin-1 at the ET_A_ receptor, has been approved for the treatment of pulmonary arterial hypertension [106]. This selective ET_A_ antagonist was developed in an effort to allow vasodilation at the same time that vasoconstriction is being targeted. The pivotal trials ARIES-1, ARIES-2 [27], and AMBITION [107] proved its long-term benefit in idiopathic and SSc-associated PAH patients.

Regarding pulmonary fibrosis, ambrisentan has not shown positive outcomes and is probably associated with an increased risk for disease progression [28], according to Raghu et al.

#### 2.6.3. Selexipag → IP Receptor Agonist

Selexipag is an oral, selective IP prostacyclin receptor agonist that has recently been approved for the long-term treatment of PAH [29]. Due to its high selectivity, its pharmacokinetic properties, and the treatment regime that is followed, selexipag is considered to have a rather reliable safety profile, with minimal adverse effects, ranging from mild to moderate in severity, considering those associated with prostacyclin use [108]. Moreover, oral selexipag has been noted to afford wide dosing flexibility, which might enable reaching the maximum therapeutic effect with acceptable tolerability in patients.

Regarding Raynaud's phenomenon, there has been no evidence that suggests that this drug has a particular effect on reducing the number of attacks [30], as shown by Denton* et al*.

#### 2.6.4. Riociguat → GMPc Agonist

Riociguat is a soluble guanylate cyclase (sGC) modulator with both vasoactive and antifibrotic effects. It is currently under evaluation for skin involvement in dcSSc patients in the RISE-SSc trial [109]. It was approved to be used for the treatment of pulmonary arterial hypertension. Its safety and efficacy were established in the PATENT studies [110]. It is important to note that, according to the PATENT-2 trial, survival of patients with PAH associated with connective tissue diseases (PAH-CTD) was similar to that seen in patients with idiopathic/familial PAH after over 2 years of treatment, which is an important observation, as mortality for PAH-CTD has been previously reported to be higher than IPAH despite modern therapy, which indicates this drug's tolerability and satisfactory clinical response.

#### 2.6.5. Bardoxolone Methyl → Nrf2 and NF-*κ*B

Bardoxolone methyl is a semisynthetic triterpenoid that upregulates antioxidant responses and suppresses proinflammatory signaling to reduce oxidative stress and inflammation and promote mitochondrial function, through activation of Nrf2 (nuclear factor erythroid derived 2-related factor 2) and inhibition of NF-*κ*B (nuclear factor kappa-light-chain-enhancer of activated B cells) [111]. It is currently being tested to treat several pathologies including pulmonary arterial hypertension, cancer, and kidney diseases [112].

Preliminary results from the extension of the LARIAT study, a phase 2 study to evaluate the safety of bardoxolone methyl in PAH patients with different causes (NCT02036970), which included some patients with PAH-CTD, showed good tolerance and sustained improvement in 6-minute walk test (6MWT) for up to 32 weeks [113].

CATALYST is a phase 3, double-blinded, placebo-controlled study to assess the safety and efficacy of bardoxolone methyl relative to placebo in patients with connective tissue disease-associated pulmonary arterial hypertension, to determine the change from baseline in 6-minute walk distance (6MWD) following 24 weeks of study participation (NCT02657356). This study started in October of 2016 and it is still active; it should be completed by mid-2020.

## 3. Conclusions

Systemic sclerosis is an autoimmune disease of unknown etiology. There are still many questions in its pathogenesis, particularly in the complex regulation of inflammatory and fibrotic processes, and in the factors that trigger its onset. Research efforts in this regard will allow finding more effective treatments, directed against therapeutic targets suitable for the different phases and complications of this condition.

## Figures and Tables

**Table 1:** Therapy proposals directed towards different aspects and molecules involved in the pathogenesis of systemic sclerosis.

Proposed therapy	Target
VASCULAR	
(i) Bosentan, macitentan	ET_A_/ET_a_ receptor
(ii) Ambrisentan	ET_A_ receptor
(iii) Selexipag	IP receptor agonist
(iv) Riociguat	GMPc agonist
(v) Methyl bardoxolone	Nrf2 and NF-kB

INFLAMMATION	
(i) Anifrolumab	Type I IFN
(ii) Sifalimumab, Rontalizumab	Type I IFN
(iii) MEDI7734	Anti-ILT7
(iv) Rituximab	CD20
(v) Basiliximab	IL-2R*α*
(vi) Efalizumab	LFA1/ICAM-1
(vii) Abatacept	CTLA4
(viii) AIMSPRO_( ® )_	*α*MSH, IL10, CCL2
(ix) Tocilizumab	IL-6R
(x) AM095, SAR100842	LPA_1_
(xi) TAK242	TLR4
(xii) Inebilizumab	Anti-CD19

FIBROSIS	
(i) Imatinib, Dasatinib, Nilotinib	c-Abl, c-Kit, PDGF
(ii) CAT-192	TGF*β*1
(iii) GC-1008	TGF*β*1,-*β*2,-*β*3
(iv) FG-3019	CCN2
(v) P144	TGF*β*1
(vi) Anti-Integrin *α*V*β*6	TGF*β* activation
(vii) Pirfenidone	TNF*α*, IL1*β*, TGF*β*
(viii) Nintedanib	VEGF, PDGF, FGF

Note: ET, endothelin; IP, G protein-coupled receptor; cGMP, cyclic guanosine monophosphate; TNF-*α*, tumor necrosis factor alpha; IL, interleukin; CCR2, chemokine receptor type 2; LFA1, lymphocyte function-associated antigen 1; ICAM-1, intercellular adhesion molecule 1; CTLA-4, cytotoxic T-lymphocyte antigen 4; *α*MSH, alpha-melanocyte stimulating hormone; CCL2, chemokine (C-C motif) ligand 2; LPA1, lysophosphatidic acid 1; c-Abl, cellular oncogene homologous to Abelson's murine leukemia; c-Kit, proto-oncogene tyrosine kinase; PDGF, platelet-derived growth factor; TGF, transforming growth factor; CCN2, type 2 connective tissue growth factor; VEGF, vascular endothelial growth factor; FGF, fibroblast growth factor; Nrf2: nuclear factor erythroid derived 2-related factor 2; NF-kB: Nuclear factor kappa-light-chain-enhancer of activated B cells.

**Table 2:** Overview of the main clinical trials that evaluate treatments against the inflammatory, fibrotic, and vascular components of SSc.

**Drug name**	**Target**	**Mechanism of action**	**Trial ID/reference**
**Treatments against immune mediators**			

**Anifrolumab** **(MEDI546)**	Directed against subunit 1 of type I interferon receptor	Downregulation of T-cell activation	NCT00930683

**Inebilizumab (MEDI551)**	Anti-CD19	Depletion of B cells through enhanced antibody-dependent cellular cytotoxicity	NCT00946699

**Sifalimumab** **Rontalizumab**	Anti-IFN*α*	Specific for IFN*α* blocking; doesn't neutralize other type I IFNs	NCT01283139
			NCT00541749

**MEDI7734**	Anti-ILT7	Temporary depletion of plasmacytoid dendritic cells	NCT02780674

**Rituximab**	Anti-CD20	Targets CD20 expressed from pre-B cell stage to the pre-plasma cell stage	Giuggioli D et al. [10]

**Basiliximab**	Anti-IL-2R*α*	Directed against the *α* chain (CD25) of the IL-2 receptor	Becker MO et al. [11]

**Efalizumab**	LFA1/ICAM-1	Interaction between LFA-1 and ICAM-1 is blocked, preventing T-cell's activation	Zimmerman T et al. [12]

**Abatacept**	CTLA4	Inhibits T-cell activation by selectively modulating costimulation (binds to CD80 or CD86 on cell surface)	Elhai M et al. [13]

**AIMSPRO (**®**)**	*α*MSH, IL10, CCL2	Modulates serum levels of relevant cytokines	Quillinan NP et al. [14]

**Tocilizumab**	IL-6R	Regulatory effect in the balance between Th17 and Tregs	NCT01532869

**AM095 and SAR100842**	LPA1	Targets specific G-protein-coupled receptors	NCT01651143

**TAK242**	TLR4	Prevents Th1 and Th17 cytokines production by inhibiting TLR4 stimulation	Bhattacharyya S et al. [15]

**Treatments against Fibrosis**			

**Imatinib**	PDGF, TGF-*β*	Blocks both PDGF and TGF-*β* signalling pathways	Iwamoto N et al. [16]

**Dasatinib,** **Nilotinib**	c-Abl, PDGF	Second-generation TKIs with higher affinity to Bcr-Abl	Akhmetshina A et al. [17]

**Metelimumab (CAT-192)**	TGF*β*1	Specifically counteracts the TGF*β*1 isoform	Denton CP et al. [18]

**Fresolimumab (GC-1008)**	TGF*β*1,-*β*2,-*β*3	Targets all isoforms of TGF*β*	NCT01284322

**FG-3019**	CCN2	Anti-CTGF, reduces the number of CD45-positive cells	Brenner MC et al. [19]

**P144**	TGF*β*1	Blocks the interaction between TGF*β*1 and TGF*β*1 type III receptor	NCT00574613, Postlethwaite AE et al. [20]

**Anti-Integrin ** **α** **V** **β** **6 (Abituzumab)**	TGF*β*	Inhibits binding to *α*v heterodimers, preventing TGF-*β* activation	NCT02745145, Katsumoto et al. [17], Henderson NC et al. [21]

**Pirfenidone**	TNF*α*, IL1*β*, TGF*β*	Blocks TGF-*β*-stimulated collagen synthesis	Udwadia ZF et al. [22] Khanna D et al. [23]

**Nintedanib** **(BIBF 1120)**	VEGF, PDGF, FGF	TKI-targeting FGF, PDGF, and VEGF receptors, as well as Src-family tyrosine kinases	NCT02597933, Distler et al. [24]

**Treatments against vascular alterations**			

**Bosentan,** **Macitentan**	ETA/ETB receptor	Blocks both ETA and ETB endothelin receptors that mediate the effects of ET-1	NCT00077584, Matucci-Cerinic M et al. [25] NCT00660179, Metha S et al. [26]

**Ambrisentan**	ETA receptor	Blocks the action of endothelin-1 at the ETA receptor	NCT00091598, NCT01178073, Gailè N et al. [27], Raghu G et al. [28]

**Selexipag**	IP receptor	Selective IP prostacyclin receptor agonist for long-term treatment of PAH	Sitbon O et al. [29], Denton CP et al. [30]

**Riociguat**	GMPc agonist	Soluble guanylate cyclase (sGC) modulator with both vasoactive and antifibrotic effects	NCT02283762, NCT00863681

**Bardoxolone methyl**	Nrf2 and NF-*κ*B	Activation of Nrf2 and inhibition of NF-*κ*B	NCT02036970, NCT02657356
